# A Novel Injection Protocol Using Voluven®-Assisted Indocyanine Green with Improved Near-Infrared Fluorescence Guidance in Breast Cancer Sentinel Lymph Node Mapping—A Translational Study

**DOI:** 10.1245/s10434-023-14129-4

**Published:** 2023-08-21

**Authors:** Yung-Chun Hsieh, Kai-Wei Guo, Man-Wen Wang, Shih-Po Su, Yu-Han Syu, Chiun-Sheng Huang, Yang-Hsiang Chan

**Affiliations:** 1 Department of Surgery, National Taiwan University Hospital Hsinchu Branch, Hsinchu, Taiwan, ROC; 2 Department of Surgery, National Taiwan University Hospital, Taipei, Taiwan, ROC; 3 National Taiwan University College of Medicine, Taipei, Taiwan, ROC; 4 Department of Applied Chemistry, National Yang Ming Chiao Tung University, Hsinchu, Taiwan, ROC; 5 Institute of Biomedical Engineering, National Yang Ming Chiao Tung University, Taipei, Taiwan, ROC; 6 Center for Emergent Functional Matter Science, National Yang Ming Chiao Tung University, Hsinchu, Taiwan, ROC; 7 Department of Medicinal and Applied Chemistry, Kaohsiung Medical University, Kaohsiung, Taiwan, ROC

**Keywords:** Near-infrared, Fluorescence-guided surgery, Indocyanine green, Sentinel lymph node biopsy, Breast cancer, 6% hydroxyethyl starch, Pharmacokinetics

## Abstract

**Background:**

Near-infrared (NIR) fluorescence-guided surgery with indocyanine green (ICG) has been demonstrated to provide high sensitivity in sentinel lymph node biopsy (SLNB) for breast cancer but has several limitations, such as unstable pharmacokinetics, limited fluorescence brightness, and undesired diffusion to neighboring tissues. This paper investigates the use of Voluven® as the solvent for ICG fluorescence-guided SLNB (ICG-SLNB).

**Methods:**

The photophysical properties of ICG in water and Voluven® were evaluated in laboratory experiments and in a mouse model. Nine patients with early breast cancer underwent subareolar injection of diluted ICG (0.25 mg/ml) for ICG-SLNB. Six of the nine patients received ICG dissolved in Voluven® (ICG:Voluven®), while three were administered ICG dissolved in water (ICG:water); a repetitive injection-observation protocol was followed for all patients. The mapping image quality was evaluated.

**Results:**

Laboratory experiments and in vivo mouse study showed improved fluorescence and better targeting using Voluven® as the solvent. ICG-SLNB with a repetitive injection-observation protocol was successfully performed in all nine patients. ICG:Voluven® administration had an overall better signal-to-background ratio (SBR) in sequential sentinel lymph nodes. The rates of transportation within the lymphatics were also improved using ICG:Voluven® compared with ICG:water.

**Conclusions:**

From basic research to animal models to in-human trial, our study proposes a repetitive injection-observation technique with ICG:Voluven®, which is characterized by better transportation and more stable mapping quality for ICG-SLNB in breast cancer patients.

**Supplementary Information:**

The online version contains supplementary material available at 10.1245/s10434-023-14129-4.

Sentinel lymph node biopsy (SLNB) is currently the standard of care for early breast cancer surgery. The status of sentinel lymph nodes (SLNs) is one of the most powerful outcome predictors of the disease. Modern SLN mapping techniques include the administration of blue dyes (e.g., patent blue, methylene blue, or isosulfan blue), radioactive colloids (e.g., ^99m^Tc-sulfur colloid or ^99m^Tc-phytate) or a combination of both, which serves as the gold standard. For blue dye, the invisibility of the SLN deep within the axilla requires substantial exploration of the wound and a longer learning curve. On the other hand, the limited accessibility and higher cost of radiocolloids, coupled with the potential radiation exposure for healthcare professionals, pose obstacles to the adoption of radioisotope-based SLN mapping methods. The lack of real-time imaging capability also makes the procedure difficult to standardize.

Given the aforementioned concerns, ICG was first used as a dye in 1999 and as a near-infrared (NIR) fluorescence imaging agent in 2005 in SLNB for breast cancer.^1,2^ ICG fluorescence-guided SLNB (ICG-SLNB) has advantages such as precise localization of the skin incision site, easy tracking of lymphatic vessels to the axillary lymph nodes, high identification rates of the SLNs and low incidence of adverse reactions.^3–5^ The high consistency of ICG and radioisotopes in SLN detection guarantees the oncological safety of this technique.^6^ In a recent meta-analysis that compared ICG-SLNB with radioisotope and blue dye-guided SLNB in breast cancer surgery, ICG-SLNB was not inferior to the dual tracer technique (combined radioisotope and blue dye) or radioisotope alone but was superior to blue dye alone.^7^

Despite the advancements made in ICG-SLNB for breast cancer in recent years, the following crucial limitations associated with this technique need to be addressed: (1) aggregation-caused quenching (ACQ) emission of ICG^8^—the ICG molecules tend to aggregate, resulting in reduced fluorescence emission; (2) diffusion of injected ICG into adjacent tissues—ICG can slowly diffuse into surrounding tissues such as subcutaneous fatty tissue if the procedure exceeds a duration of 30 min, leading to increased background noise levels;^2,9^ and (3) unstable pharmacokinetics of ICG in lymphatic vessels—the rate of ICG transportation in lymphatic vessels is unstable.^10^ These fluctuations in the transportation rate can undermine its effectiveness as a tracer and compromise surgeons’ confidence. As a result, operating room lights often need to be turned off, which is inconvenient and raises safety concerns during surgeries. Additionally, the lack of optimization and standardization in the administration of ICG poses limitations in the development of medical NIR imaging devices.^11,12^

To overcome these challenges, we propose a bold approach that involves substituting distilled water, the stock solvent for ICG (Diagnogreen®), with Voluven®. Voluven® is a synthetic colloid composed of 6% hydroxyethyl starch (HES) 130/0.4 in 0.9% sodium chloride, primarily used for plasma volume replacement to restore blood volume. This is the first study to utilize Voluven® as the solvent for ICG in fluorescence image-guided surgery. The rationale for the use of Voluven® lies in the hydrophobic interactions between the HES colloids present in Voluven® and ICG.^13^ By leveraging these hydrophobic interactions, the ACQ effect can be alleviated, thereby enhancing the fluorescence emission of ICG. Furthermore, we hypothesized that these hydrophobic interactions can help confine the injected ICG within the lymphatic vessels, minimizing diffusion into adjacent tissues and reducing background noise levels. The colloidal properties of Voluven® may facilitate improved pharmacokinetics, stabilizing the transportation of ICG through lymphatic vessels.^14,15^

To prove the proposed concept, we first fundamentally analyzed the photophysical properties of ICG in saline, Voluven®, and human serum albumin (HSA) at different concentrations. Further evaluation of ICG in Voluven® (ICG:Voluven®) compared with ICG in water (ICG:water) in mice was performed. The optimal conditions were then clinically translated for ICG-SLNB in breast cancer patients. The goal of this comprehensive approach was to bridge the gap between fundamental research and clinical application, ensuring that the proposed concept underwent rigorous validation in the field of translational medicine.

## Methods

### Preparation of ICG Solutions

**Fig. 1:**
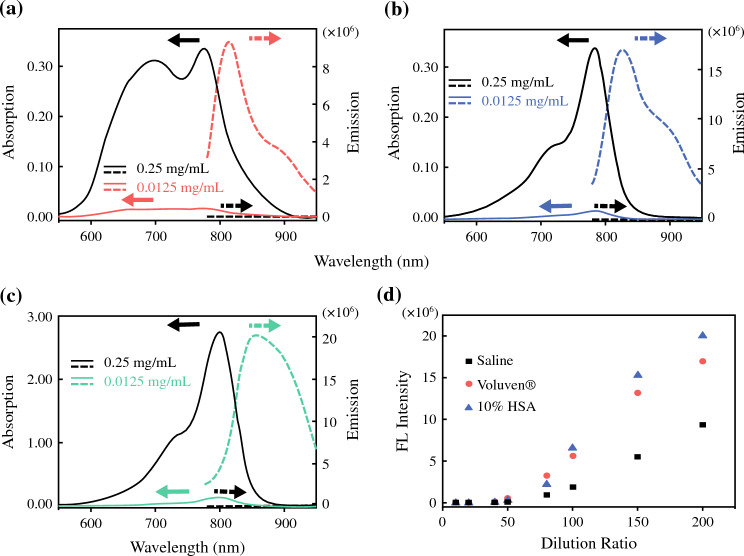
(**a**) Absorption (*solid lines*) and emission (*dashed lines*) of ICG in saline at concentrations of 0.25 mg/ml (*black lines*) and 0.0125 mg/ml (*red lines*). (**b**) Absorption (*solid lines*) and emission (*dashed lines*) of ICG in Voluven® at concentrations of 0.25 mg/ml (*black lines*) and 0.0125 mg/ml (*blue lines*). (**c**) Absorption (*solid lines*) and emission (*dashed lines*) of ICG in 10% HSA at concentrations of 0.25 mg/ml (*black lines*) and 0.0125 mg/ml (*green lines*). (**d**) Concentration-dependent fluorescence trends of ICG in saline, Voluven®, and 10% HSA

All reagents were purchased from Sigma‒Aldrich, Alfa Asear, TCI, and Thermo Fisher and used as received unless indicated elsewhere. Highly pure water (18.2 MΩ·cm) was used in all of the experiments. ICG for injection (25 mg/vial) was purchased from Daiichi Sankyo Propharma Co. Ltd. (Japan). Voluven® (1000 ml/bag) was obtained from Fresenius Kabi Deutschland GmbH (Germany). Flexbumin human serum albumin USP (25% solution, 50 ml/bag) was purchased from Bazalta US Inc. (IL, USA). For absorption and fluorescence measurements, three stock solutions were prepared: (1) an ICG solution with a concentration of 2.5 mg/ml using normal saline as the solvent, (2) an ICG solution with a concentration of 2.5 mg/ml using Voluven® as the solvent, and (3) an ICG solution with a concentration of 2.5 mg/ml using 10% HSA as the solvent. The ICG solutions were diluted in the three different solvents to obtain ICG solutions with concentrations of 0.25, 0.125, 0.0625, 0.05, 0.0313, 0.025, 0.0167, and 0.0125 mg/ml. This allowed measurement of the absorbance and fluorescence intensity (Fig. 1).

### In Vivo NIR Imaging in Mice (See Supplementary Information for more details)

To investigate the in vivo NIR fluorescence imaging properties of ICG:Voluven® and ICG:water, three nude mice were intravenously administered ICG:Voluven® and three were administered ICG:water. Subsequently, the mice were simultaneously imaged using an NIR InGaAs camera under 793 nm laser excitation (exposure time: 1000 ms, 1300 nm filter) for up to 24 h after contrast injection (*n* = 3 in each group). In vivo NIR imaging was performed to visualize whole-body blood vessels and the tumor region.

### Clinical Trial Study Design

The clinical trial was approved by the Institutional Review Board (IRB) of National Taiwan University Hospital Hsin-Chu Branch in accordance with the ethical standards of the Helsinki Declaration of 1975. It was designed as a dose-adjustment trial and was registered at clinicaltrial.gov prior to the recruitment of the participants (NCT05365204). The off-label use of ICG and Voluven® for breast cancer SLN mapping was evaluated and approved by the IRB. All participants were Taiwanese women with early breast cancer eligible for SLNB who provided written informed consent before the surgery. The exclusion criteria were known allergy to conventional tracers, known allergy to ICG, or any change of the disease resulting in the patient no longer being indicated for SLNB. The trial included a feasibility test (phase I) of using ICG:Voluven® for breast cancer SLNB, and a dose-adjustment trial for best imaging quality (phase II). The default ICG:Voluven® concentration of 0.25 mg/ml was selected based on the results from laboratory experiments and animal models. We stepwisely tested each new concentration with a group of three patients. After each group of three patients was tested, we performed image analysis. The new concentration group was stopped if the outcome (see the Section “Endpoints” below) was significantly worse than the previous concentration group. This translational research is the result of the phase I study.

### The Repetitive Injection-Observation Protocol

**Fig. 2:**
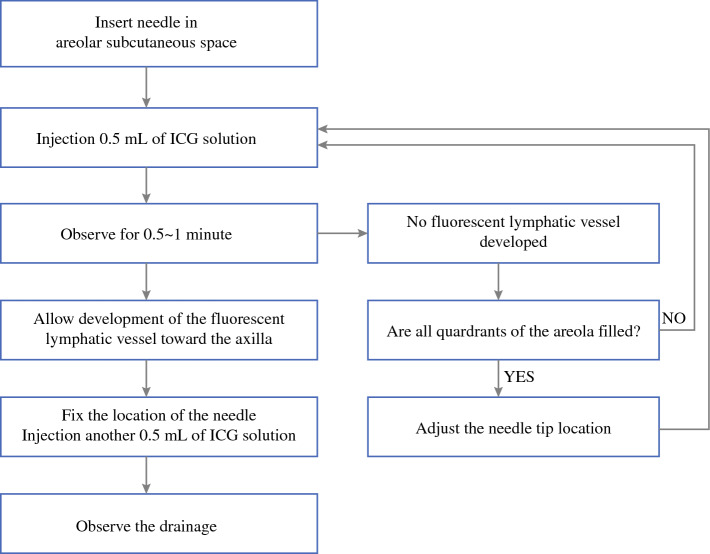
Flowchart detailing the repetitive injection-observation protocol

**Fig. 3:**
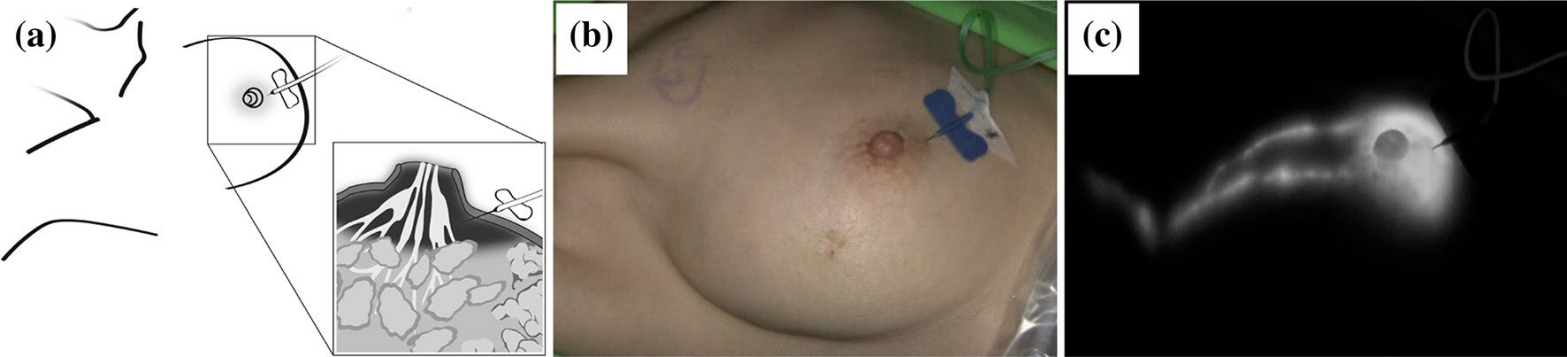
(**a**) Schematic diagram illustrating the needle placement of the winged infusion set. (**b**) Bright view showing a 66-year-old woman undergoing SLNB with the new protocol (ICG:Voluven®, 0.25 mg/ml). The needle of the winged infusion set was inserted subcutaneously beneath the areola. (**c**) The corresponding ICG fluorescence image obtained by the Stryker SPY-PHI NIR imaging system

During the surgical procedure, a repetitive injection-observation protocol was implemented (see Fig. 2). A 5-ml syringe was filled with the prepared ICG solution and connected to a winged infusion set. The needle of the winged infusion set delivered a subareolar injection (see Fig. 3). With the Stryker SPY-PHI (Stryker Corporation, Kalamazoo, MI) portable handheld NIR imaging system, 0.5 ml of the ICG solution was infused slowly. The filling of the solution into the areola and drainage to the subcutaneous lymphatic system was observed. If the whole areola was filled and the lymphatic duct traveling toward the axilla was observed, the winged infusion set was fixed in place using sterile 3M tape. If the lymphatic duct traveling toward the axilla was not observed within 2 min, the needle tip position was adjusted to another quadrant by the surgeon. The injection and observation were repeated. Light palpation of the breast was performed if draining seemed stationary. Injection example videos are provided in the supplementary files (see Vid. S1 for ICG:Voluven® and Vid. S2 for ICG:water).

### Conventional Tracer Selection and the Sentinel Lymph Node Biopsy Procedure

Given that ICG is not yet an FDA-approved dye for breast cancer SLN mapping, an FDA-approved primary tracer was used, forming a dual-tracer mapping technique. Since the study goal was to evaluate the performance of the Voluven®-assisted ICG solution in SLN mapping of breast cancer, we carefully avoided synchronous injection of multiple tracers at the same location to prevent interaction between the drugs affecting the outcome evaluation. Peritumoral blue dye (Patent Blue V®) was selected as the primary (conventional) tracer for SLN mapping.

We usually waited for at least 5 min after blue dye injection and then initiated the SLNB procedure. All blue nodes were removed. ICG-fluorescent SLNs were retrieved sequentially, guided by the fluorescent lymphatic ducts. It is a known fact that ICG fluorescence guided SLN biopsy tends to retrieve more nodes than the conventional methods, and there are considerations of over-extensive dissection to the axilla.^7,16^ Therefore, during the procedure, the first three fluorescent nodes receiving drainage from the breast were retrieved. If additional sequential nodes were observed during NIR fluorescence imaging, the first three nodes were sent for frozen section analysis. Further dissection was not carried out if the frozen section was reported to be benign.

### Measurement of Signal-to-Background Ratio (SBR)

All lymph node images were taken using the SPY-PHI NIR fluorescence mode (grayscale SPY mode) with a medical HDMI video recorder. The SBR values of the lymph nodes were measured by ImageJ bundled with Java 1.8.0_172 software. R version 4.2.2 (R Foundation, Vienna, Austria) was used for plotting.

### Endpoints

The primary endpoint was the number of retrieved lymph nodes and SBR under the SPY-PHI imaging system. Other endpoints included the areola-to-axilla traveling time (AAT) of ICG fluorescent lymphatics (the time from the development of subcutaneous lymphatic fluorescence to the fluorescence reaches the axillary fascia), the status of blue dye and ICG fluorescence in retrieved SLNs, and any adverse effects of this injection procedure.

## Results

### Effect of ICG Concentration on Optical Properties

We measured the absorption and fluorescence spectra of ICG at concentrations of 0.25 mg/ml and 0.0125 mg/ml in various environments, including saline, Voluven®, and 10% HSA (Fig. 1a–c). These experimental findings offered insights into the ACQ effect of ICG and its emission intensities, which are concentration-dependent in different environments. Additionally, we investigated the dilution ratio factor of the three stock solutions (0.25 mg/ml), as depicted in Fig. 1d. Table 1 provides a summary of the fluorescence intensities for each data point depicted in Fig. 1d.

**Table 1:** Emission intensities of ICG with different dilution ratios in three different solutions

Concentration (mg/ml)	Dilution ratio	Saline solution	Voluven® solution	10% HSA solution
0.2500	10×	9.26 × 10^1^	9.85 × 10^1^	7.88 × 10^2^
0.1250	20×	2.11 × 10^2^	5.08 × 10^2^	3.98 × 10^3^
0.0625	40×	1.58 × 10^4^	1.35 × 10^5^	4.28 × 10^4^
0.0500	50×	6.92 × 10^4^	5.10 × 10^5^	2.41 × 10^5^
0.0313	80×	9.27 × 10^5^	3.25 × 10^6^	2.17 × 10^6^
0.0250	100×	1.88 × 10^6^	5.60 × 10^6^	6.61 × 10^6^
0.0167	150×	5.52 × 10^6^	1.32 × 10^7^	1.53 × 10^7^
0.0125	200×	9.35 × 10^6^	1.69 × 10^7^	2.00 × 10^7^

### In Vivo NIR Imaging in Mice

Fluorescence imaging of ICG:Voluven® and ICG:saline in Eppendorf tubes was conducted using a 1300-nm longpass filter and an exposure time of 500 ms (Fig. S1a). Clear visualization of whole-body blood vessels was achieved with NIR imaging with ICG:Voluven® as well as ICG:water (Fig. S1b, see Supplementary Information, SI) within 1 min of injection. In the in vivo imaging in mice (Fig. S1b and c), ICG was intravenously injected into live mice, bearing 4T1 tumors, through the tail vein. ICG:Voluven® and ICG:water were administered to three mice each. Within 1 min of the injection, ICG dispersed throughout the entire body, enabling clear visualization of the vascular structures in the live mice. As early as 1 min postinjection, ICG uptake in the liver, kidney, and vessels was detected. However, ICG:Voluven® provided greater vessel conspicuity and enabled prolonged visualization of vasculature for up to 5 min, while with ICG:water, blood vessels were not visualized beyond 2 min. The signals from background soft tissues and the liver gradually decreased due to quick elimination, accompanied by increasing signals in the tumor. Furthermore, the SBR was consistently higher for ICG:Voluven® than for ICG:water at all time points (Fig. S1c). At 1 min postinjection, the SBR values were 1.58 for ICG:Voluven® (Fig. S1d) compared with 1.43 for ICG:water (Fig. S1e). At 5 min postinjection, the SBR of ICG:water dropped to less than 1, while that of ICG:Voluven® remained higher at 1.35. After 24 h, the mice were sacrificed, and isolated tissues, including those of the pancreas, liver, spleen, kidney, and tumor, were collected (Fig. S1f and g). ICG fluorescence was predominantly concentrated in the kidneys, and stronger fluorescence was observed in tumors from mice treated with ICG:Voluven® compared with ICG:water.

### Indocyanine Green NIR Fluorescence-Guided Sentinel Lymph Node Biopsy in Nine Patients

In total, nine patients undergoing SLNB for breast cancer were enrolled from April 2022 to August 2022. Six patients underwent a repetitive injection-observation protocol using ICG:Voluven® (0.25 mg/ml). Three patients were administered ICG:water (0.25 mg/ml) for comparison. The patient characteristics and information about tumor typing and tumor staging are shown in Table 2. All patients were female, and their ages ranged from 34 to 80 years. Their BMIs ranged from 15.1 to 34.9. The invasive tumors were T1 and T2 lesions, ranging in size from 8 to 32 mm. One patient had one SLN pathologically positive for metastasis.

**Table 2:** Summary of patient characteristics

Case	Age	BMI	Side	Tumor size (mm)	Type of invasive carcinoma	Grade	ER	PR	HER-2 score	Ki-67 index	Pathological staging
V1	64	19.6	Left	10	NST	I	> 95%	> 95%	0/3+	10–15%	pT1b pN0
V2	36	20.9	Right	20	Mixed lobular and NST	II	> 95%	> 95%	0/3+	5–30%	pT1c pN1a
V3	80	22.2	Left	9	Solid papillary (multifoci)	II	> 95%	> 95%	0/3+	15–20%	mpT1b pN0
V4	43	23.8	Left	8	Tubular	I	> 95%	> 95%	1+/3+	10–20%	pT1b pN0
V5	65	15.1	Right	22	NST	III	> 95%	30%	1+/3+	40–70%	pT2 pN0
V6	50	34.9	Right	28	NST	III	3%	0%	1+/3+	60–80%	pT2 pN0
W1	34	20.6	Left	18	NST	II	> 95%	80	1+/3+	15–60%	pT1c pN0
W2	60	27.1	Right	32	NST	III	3%	< 1%	0/3+	60–90%	pT2 pN0
W3	36	19.5	Left	16	NST	III	95%	30%	2+/3+(FISH negative)	50–70%	pT1c pN0

*BMI*, body mass index; *ER*, estrogen receptor; *PR*, progesterone receptor; *NST*, no special type; *HER-2*, human epidermal growth factor receptor 2; *W*, ICG:water-treated patient; *V*, ICG:Voluven®-treated patient.

**Fig. 4:**
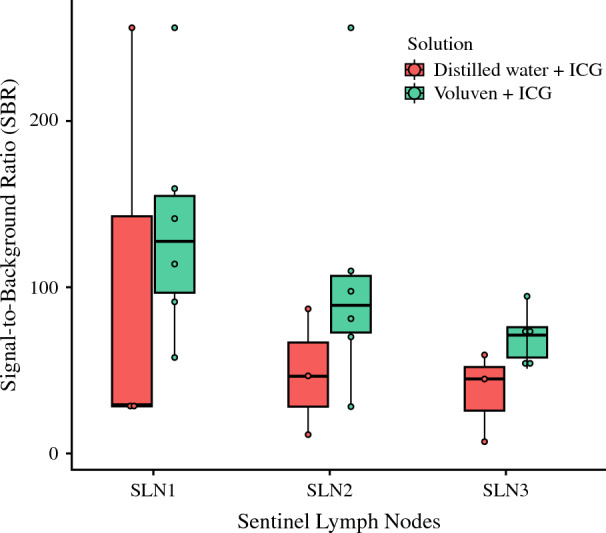
The SBRs of the retrieved SLNs. The SBRs of the “overexposed” SLNs were not evaluable on the 8-bit grayscale images (>255:1); thus, they were coded as “256” in this figure for better visualization. The actual contrast ratio could be higher

All patients had lymphatic vessels successfully identified under NIR fluorescence. No adverse reactions were observed intraoperatively or reported by patients postoperatively. The type and volume of injected solution, AAT, measured SBRs, and pathological status of the retrieved SLNs are all listed in Table 3. The SBRs of the retrieved nodes were measured (Fig. S2 as an example) and are plotted in Fig. 4. The volume of the injected solution ranged from 1.5 to 2.5 ml. The AAT ranged from 163 to 196 s for ICG:water and 21 to 133 s for ICG:Voluven®. Except for one patient (V2) with metastasis detected in the first SLN, three SLNs were retrieved from all other patients. In the case of patient V1, the needle tip was inadvertently inserted too deeply into the breast parenchyma, leading to leakage of the ICG solution from the nipple (Fig. S3). The mapping of the SLNs was not affected after adjustment of the needle tip position. One patient (W1) had no blue nodes identified. As per the trial design, the three ICG:water participants presented obviously worse results in the endpoints (lymph node SBRs and AATs) compared with ICG:Voluven®. Thus, no more participants were subsequently invited into the ICG:water group.

**Table 3:** Sentinel lymph node status by ICG-SLNB in 9 patients

Case	Solution^a^	Volume (ml)	AAT (s)	SLN1			SLN2			SLN3		
				SBR	Blue	Status	SBR	Blue	Status	SBR	Blue	Status
V1	B	2	31	57.6	Yes	Benign	97.2	No	Benign	75.7	No	Benign
V2	B	1.5	21	90.9	Yes	Metastatic	27.9	No	Benign		(N/A)	
V3	B	2	56	>255	Yes	Benign	>255	Yes	Benign	50.7	No	Benign
V4	B	2	97	159.2	Yes	Benign	70.1	Yes	Benign	94.3	No	Benign
V5	B	2	133	141.2	Yes	Benign	80.8	No	Benign	57.6	No	Benign
V6	B	2.5	96	113.6	Yes	Benign	109.8	Yes	Benign	70.9	No	Benign
W1^b^	A	2	163	27.7	No	Benign	86.5	No	Benign	44.6	No	Benign
W2	A	2	196	29.3	No	Benign	46.4	Yes	Benign	59.3	No	Benign
W3	A	2	141	>255	No	Benign	11.4	Yes	Benign	6.9	No	Benign

^a^Solution *A* = 0.25 mg ICG in 1 ml water; Solution *B* = 0.25 mg ICG in 1 ml Voluven®.

^b^Case W1 has two draining lymphatics toward the axilla. No blue node was found. The SLN1 draining route is different from the routes for SLN2 and SLN3.

*AAT*, areola-to-axilla traveling time; *SLN*, sentinel lymph node; *BMI*, body mass index; *OE*, overexposure; *N/A*, not applicable.

## Discussion

ICG-SLNB has been performed in breast cancer patients for over a decade. This procedure has garnered interest among oncologic surgeons due to its numerous advantages, including low toxicity, real-time evaluation, intraoperative adjustability, and high sensitivity. However, the optimization and standardization of the injection technique have not been well established, resulting in variations in the reproducibility of the procedure across different studies. In 2014, Ahmed et al.^16^ reviewed available studies and reported the absence of visible lymphatics during ICG-SLNB in between 0 and 43% of studies. Moreover, the reviewed studies reported variations in the injection technique, including different concentrations, injection locations, and solvents used. For example, Kitai et al.^2^ used 25 mg/5 ml ICG:water for subareolar injections, Troyan et al.^3^ used 10 μM ICG:HSA solution for multipoint peritumoral injections, and Inoue et al.^17^ used a mixed solution of ICG and patent blue (1.0 ml of 0.5% ICG mixed with 2.0 ml of 1% patent blue) for subareolar injection. These mixing techniques are intended to address a common challenge encountered in ICG fluorescence lymphatic mapping, which is the instability of ICG pharmacokinetics from the time of injection to its final transportation into the target object. Due to the visible drainage of lymphatics and the ability to adjust the ICG solution during surgery, the total injection volume is often determined based on the surgeon’s experience.^6^

### Effect of ICG Concentration on Optical Properties

We observed a significant adverse ACQ effect of ICG at concentrations exceeding 0.25 mg/ml, as evidenced by very low fluorescence intensities across all three different solvents (Fig. 1 and Table 1). Even under such concentrated conditions, the fluorescence of ICG in 10% HSA exhibited 8–8.5 times higher intensity compared with saline and Voluven®. This phenomenon can be attributed to the noncovalent binding between the dye and proteins, facilitated by hydrophobic van der Waals interactions as well as ion pairing between the sulfonate groups of ICG and cationic amino acid residues such as histidine, lysine, and arginine.^18,19^ Similar behavior has been observed for cyanine derivatives with sulfonate moieties in solutions containing serum.^20^ The mechanism underlying this phenomenon involves the hindrance of dye aggregation by protein binding, thus maintaining a rigid dye conformation that minimizes torsional effects and, consequently, reduces nonradiative decay. When the concentration of ICG was significantly diluted to 0.0125 mg/ml, the emission intensities of ICG showed a substantial increase. Specifically, the fluorescence intensities increased by approximately 5 and 5.2 orders of magnitude for saline and Voluven®, respectively. In contrast, a smaller increase in ICG fluorescence, less than 5 orders of magnitude, was observed for 10% HSA. The observed increase in fluorescence relative to the dilution ratio (Fig. 1d) suggests that hydrophobic HES plays a similar role to HSA in relation to ICG. Notably, concentrated ICG in water exhibited significant H-aggregates, which was evident by the strong absorption shoulder at ~700 nm (Fig. 1a).^21^ On the other hand, when concentrated ICG was mixed with Voluven® and 10% HSA, a similar absorption profile was observed, suggesting the absence of aggregation (Fig. 1b and c). This finding again suggests that the HES in Voluven® effectively prevents aggregation of ICG and exhibits behavior similar to that of HSA.

### In Vivo NIR Imaging in Mice

From the brightness measurements of ICG:Voluven® and ICG:water in Eppendorf tubes, both at a concentration of 0.25 mg/ml (Fig. S1a), the emission brightness of ICG:Voluven® was ~1.5 times higher than that of ICG:water, which is consistent with the spectroscopic results (Table 1). Interestingly, we found that the SBR and the spatial resolution of blood (Fig. S1d and e) were notably improved in mice treated with ICG:Voluven® compared with those injected with ICG:water. This finding verified our proposed concept that ICG has a tendency to adhere to the HES colloids in Voluven®, consequently reducing its diffusion into neighboring tissues and minimizing background noise levels. More strikingly, from the biodistribution of ICG in major organs 24 h after injection, it was observed that ICG:Voluven® exhibited specific accumulation within the 4T1 tumors. This targeting ability could be attributed to the enhanced permeability and retention effect, a phenomenon that facilitates the accumulation of macromolecules, lipids, and nanoparticles (e.g., HES colloids) in tumor tissues.^22,23^ The performance of ICG with tumor targeting ability by Voluven® could promote the widespread adoption of ICG:Voluven® formulations in fluorescence-guided surgeries utilizing tumor targeting probes.

### ICG Fluorescence-Guided Sentinel Lymph Node Mapping

We clinically translated the hypothesis that ICG could be “protected” from the ACQ effect and unwanted binding with serum proteins in the lymphatics when dissolved in Voluven® solution. The observed shorter AAT and higher SBR of retrieved lymph nodes in this study show improved transportation of the ICG when assisted by the Voluven® solution. In 2011, a dose-image optimization trial by Mieog et al.^24^ was published, concluding that the ACQ effect at the injection site and the dilution within lymphatic channels were major contributors to the signal strength of the SLN. Our study provides a protocol with lower transportation loss, which means a better possibility for sequential mapping of more draining lymph nodes.

After the ACOSOG Z0011 trial, AMAROS trial, and SINODAR-ONE trial, current guidelines propose omitting axillary lymph node dissection when meeting certain criteria and fewer than three SLNs are metastasized.^25–28^ In 2022, Yuan et al.^29^ revealed a novel approach, stepwise limited axillary lymph node dissection, as a de-escalated form of traditional axillary lymph node dissection. This procedure enabled further mapping beyond the conventional SLNs and was reported to have a significantly lower lymphedema rate with no obvious compromise to the oncological outcomes. We assume that our repetitive injection-observation protocol with ICG:Voluven® could be an easier substitute for the stepwise blue dye injection procedure in this type of surgery. Future research could be conducted for further de-escalation of axillary surgeries in breast cancer patients.

## Conclusions

From basic research to animal models to in-human trial, our study proposes a repetitive injection-observation technique with ICG:Voluven®, which is characterized by better transportation and more stable mapping quality for ICG-SLNB. This breakthrough could promote the versatile use of ICG:Voluven® formulations in a wide range of surgical procedures for precise targeting and localization.

### Supplementary Information

Below is the link to the electronic supplementary material.

Supplementary file1 (DOCX 11658 KB)

Supplementary file2 (MP4 60340 KB)

Supplementary file3 (MP4 205253 KB)
