# Supplementary material for: A Novel Injection Protocol Using Voluven®-Assisted Indocyanine Green with Improved Near-Infrared Fluorescence Guidance in Breast Cancer Sentinel Lymph Node Mapping—A Translational Study
Source: Ann Surg Oncol. 2023 Aug 21;30(13):8419–27. doi: 10.1245/s10434-023-14129-4 (PMC10625936; doi:10.1245/s10434-023-14129-4)
Supplement: Supplementary file 1 — Supplementary file1 (DOCX 11658 KB) [file 10434_2023_14129_MOESM1_ESM.docx]

**- SUPPLEMENTARY INFORMATION -**

**A Novel Injection Protocol Using Voluven®-Assisted Indocyanine Green with Improved Near-Infrared Fluorescence Guidance in Breast Cancer Sentinel Lymph Node Mapping – A Translational Study**

Yung-Chun Hsieh M.D., MMSC^abc^, Kai-Wei Guo, MS^d^, Man-Wen Wang, MS^d^, Shih-Po Su, PhD^e^, Yu-Han Syu, BS^d^, Chiun-Sheng Huang M.D., PhD, MPH^bc^ and Yang-Hsiang Chan, PhD*^dfg^

^a^Department of Surgery, National Taiwan University Hospital, Hsinchu Branch, Hsinchu, 30010 Taiwan, R.O.C.

^b^Department of Surgery, National Taiwan University Hospital, Taipei, 10002 Taiwan, R.O.C.

^c^National Taiwan University College of Medicine, Taipei, 10051 Taiwan, R.O.C.

^d^Department of Applied Chemistry, National Yang Ming Chiao Tung University, Hsinchu, 30010 Taiwan R.O.C.

^e^Institute of Biomedical Engineering, National Yang Ming Chiao Tung University, Taipei, 11221 Taiwan, R.O.C.

^f^Center for Emergent Functional Matter Science, National Yang Ming Chiao Tung University, Hsinchu, Taiwan, R.O.C.

^g^Department of Medicinal and Applied Chemistry, Kaohsiung Medical University, Kaohsiung, 80708 Taiwan, R.O.C.

***CORRESPONDING AUTHOR**

Prof. Yang-Hsiang Chan, E-mail: yhchan@nycu.edu.tw

Methods

***In Vivo NIR Imaging System***

Ninox 640 II (Raptor Photonics, Northern Ireland) digital camera, equipped with a 640 x 512 InGaAs sensor, was employed to detect the fluorescence signals from ICG. The excitation light source was a high power (30W) fiber coupling diode CW laser system with the laser wavelength at 808±3 nm (CNI Optoelectronics Tech. Co., Ltd., P.R. China). The laser fiber head was mounted with a SMA fiber adapter cap with internal SM1 (1.035"-40) threads (Thorlabs Inc., USA) and a ground glass diffuser (DG10-120-MD - Ø1", SM1-Mounted N-BK7, Thorlabs Inc., USA) to set the output laser density below 200 mW/cm2. For image capture, the EPIX® XCAP Std software was utilized with the Ninox 640 II camera. The exposure time was set to 20-30 ms, utilizing a low gain mode for the continuous photoshoot. The signal-to-background (SBR) values were measured by ImageJ software.

***In Vivo Mouse Model***

Mice of the BALB/c nude female strain (weighing 5 to 6 weeks and 18 to 22 g) were acquired from the National Laboratory Animal Center (Taipei, Taiwan). They had free access to food and water and were kept in a temperature-controlled environment (22 ± 1°C) with a 12-h light/dark cycle. All animal studies were approved by the Institutional Animal Care and Use Committee (IACUC) of the National Yang Ming Chiao Tung University (1100509, approved date 9 May 2021).

The 4T1 triple-negative murine breast cancer cell line was acquired from the American Type Culture Collection (ATCC, Manassas, VA, USA). The cell line was cultured in RPMI medium (GIBCO® Invitrogen Inc., Carlsbad, CA, USA) supplemented with 10 % fetal bovine serum (HyClone® Thermo, Waltham, MA, USA), 50 µg/mL penicillin/streptomycin (Sigma-Aldrich Co., St. Louis, MO, USA), and 2 mM L-Glutamine (Sigma-Aldrich Co., St. Louis, MO, USA). The cells were kept at 37°C in a humidified incubator with 5% CO_2_, needing to be passaged every two days. Female nu/nu mice aged between 4 and 6 weeks were injected subcutaneously with 2 x 106 4T1 tumor cells in 100 μL of serum-free media on the right leg. Tumors were allowed to grow subcutaneously for a period of two weeks until they were of a suitable size to allow for imaging.

In vivo imaging was performed in nude mice. Induction of anesthesia was done in an induction chamber with 2% isoflurane and maintained with 1±1.5% isoflurane delivered via a nose cone setup. The tail vein was catheterized for injection of the dye. The animals were secured to a platform in the supine position, with the ventral side facing the camera and laser source. The ICG solution was made fresh and used within 3 h. Each mouse was injected with a bolus of either ICG solution in DI water (0.25 mg/mL) or ICG solution in Voluven® (0.25 mg/mL) *via* the tail vein catheter. Images were captured during injection and at various times post injection (1 min, 5 min, 10 min, 30 min, 1 h, 2 h, 4 h, 8 h, 12 h, and 24 h). The mice were sacrificed after 24 h. Our fluorescence system was used to spectrally image various organs and tissues. A biodistribution analysis was conducted by calculating the average ICG fluorescent intensity of each imaged organ.

**FIG. S1.** (A) Mean fluorescence intensities of ICG in saline, and ICG in Voluven®. The inset represents the corresponding photographs (upper panel) and NIR fluorescence images (bottom panels). (B) Real-time whole-body imaging of vascular structures in mice (*n* = 3) bearing 4T1 tumor intravenously injected by Voluven®-dissolved ICG in the prone position at certain time intervals from 1 min to 24 h. (C) Real-time whole-body imaging of vascular structures in mice (*n* = 3) bearing 4T1 tumor intravenously injected by saline-dissolved ICG in the prone position at certain time intervals from 1 min to 24 h. (D) Cross-sectional intensity profile along the red line in (B). (E) Cross-sectional intensity profile along the yellow line in (C). (F) Biodistribution of Voluven®-dissolved ICG in major excised organs at 24 h post injection in which the quantitative mean fluorescence intensities in organs were plotted. The inset shows the bright-field image (left) and NIR-II fluorescence imaging (right) of different organs. Li, liver; Int, intestinal; Sp, spleen; Pa, pancreas; Ki, kidney; St, stomach; tu, tumor; Sk, skin. The scale bars are 10 mm in B&C.

**FIG. S2.** (A) Fluorescence image of retrieved nodes from patient 2 (case V2). (B) Cross-sectional intensity profile along the white line in (A).


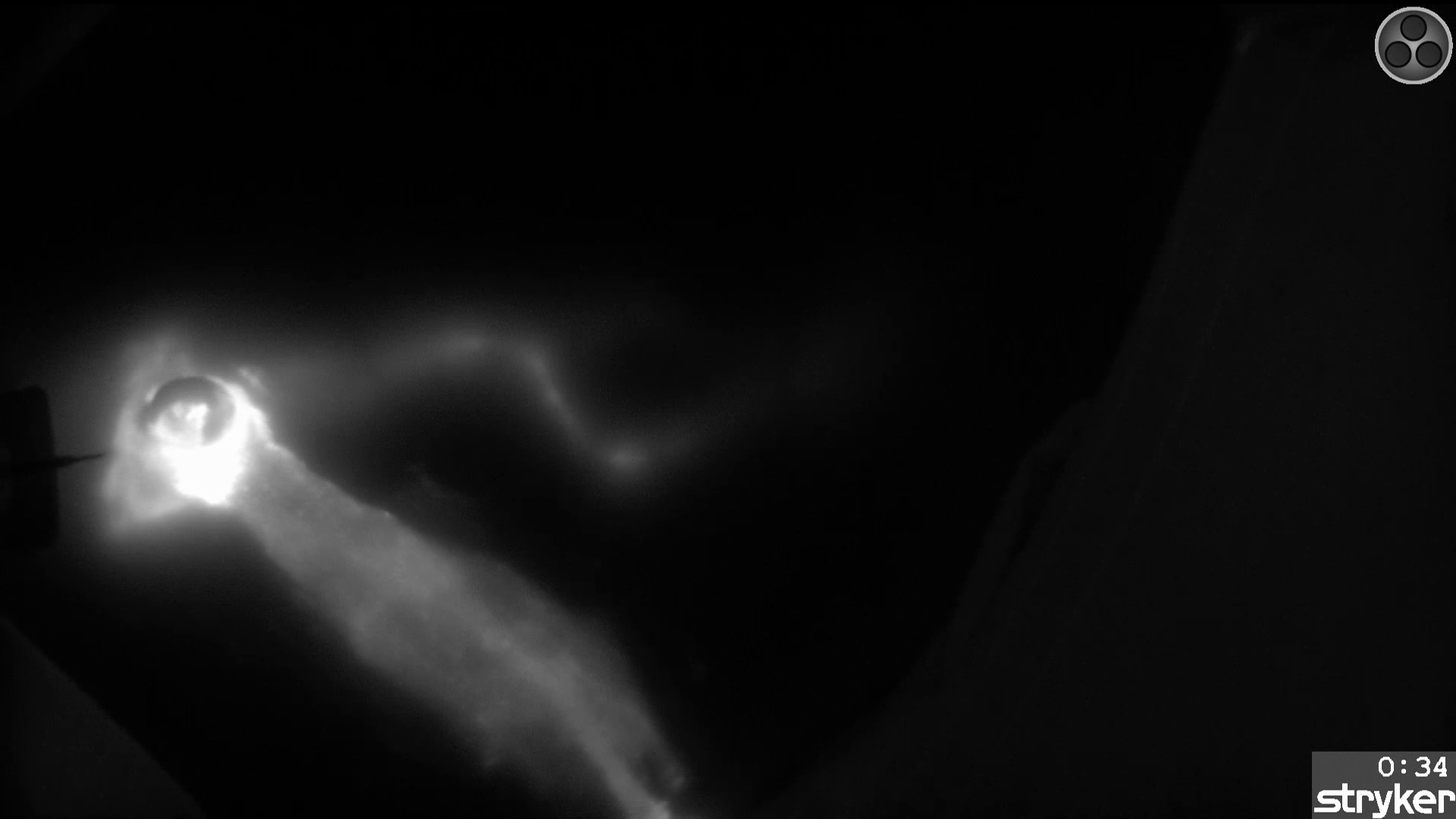


**FIG. S3.** Inadvertent leakage of the ICG solution from the nipple in patient 1 (case V1).
